# Growth Hormone and Insulin-like Growth Factor-I Molecular Weight Isoform Responses to Resistance Exercise Are Sex-Dependent

**DOI:** 10.3389/fendo.2020.00571

**Published:** 2020-08-21

**Authors:** Joseph R. Pierce, Brian J. Martin, Kevin R. Rarick, Joseph A. Alemany, Jeffery S. Staab, William J. Kraemer, Wesley C. Hymer, Bradley C. Nindl

**Affiliations:** ^1^Military Performance Division, U.S. Army Research Institute of Environmental Medicine, Natick, MA, United States; ^2^Neuromuscular Research Laboratory/Warrior Human Performance Research Center, Department of Sports Medicine and Nutrition, University of Pittsburgh, Pittsburgh, PA, United States; ^3^Department of Kinesiology, University of Connecticut, Mansfield, CT, United States; ^4^Department of Biochemistry and Molecular Biology, The Pennsylvania State University, University Park, PA, United States

**Keywords:** exercise endocrinology, sex differences, HPLC, fractionation, molecular weight variants

## Abstract

**Purpose:** To determine if acute resistance exercise-induced increases in growth hormone (GH) and insulin-like growth factor-I (IGF-I) were differentially responsive for one or more molecular weight (MW) isoforms and if these responses were sex-dependent.

**Methods:** College-aged men (*n* = 10) and women (*n* = 10) performed an acute resistance exercise test (ARET; 6 sets, 10 repetition maximum (10-RM) squat, 2-min inter-set rest). Serum aliquots from blood drawn Pre-, Mid-, and Post-ARET (0, +15, and +30-min post) were processed using High Performance Liquid Chromatography (HPLC) fractionation and pooled into 3 MW fractions (Fr.A: >60; Fr.B: 30–60; Fr.C: <30 kDa).

**Results:** We observed a hierarchy of serum protein collected among GH fractions across all time points independent of sex (Fr.C > Fr.A > Fr.B, *p* ≤ 0.03). Sex × time interactions indicated that women experienced earlier and augmented increases in all serum GH MW isoform fraction pools (*p* < 0.05); however, men demonstrated delayed and sustained GH elevations (*p* < 0.01) in all fractions through +30-min of recovery. Similarly, we observed a sex-independent hierarchy among IGF-I MW fraction pools (Fr.A > Fr.B > Fr.C, *p* ≤ 0.01). Furthermore, we observed increases in IGF-I Fr. A (ternary complexes) in men only (*p* ≤ 0.05), and increases in Fr.C (free/unbound IGF-I) in women only (*p* ≤ 0.05) vs. baseline, respectively.

**Conclusions:** These data indicate that the processing of GH and IGF-I isoforms from the somatotrophs and hepatocytes are differential in their response to strenuous resistance exercise and reflect both temporal and sex-related differences.

## Introduction

Resistance exercise is a potent stimulus, perturbing homeostasis and promoting both favorable physiological and metabolic adaptations (1). The associated responses to resistance exercise involve numerous signaling pathways and are speculated to be partially mediated by the release of growth hormone (GH) and insulin-like growth factor-I (IGF-I) (2). GH is a polypeptide hormone secreted by the anterior pituitary gland, which contributes to multiple biological processes, such as anabolism, protein synthesis, and substrate mobilization (3, 4). An explanation for such pleiotropic nature is that GH exists as a family of more than 100 isoforms (5) differing in biological and immunological activity (6). The metabolic and anabolic responses associated with GH are mediated via the interaction of GH with the GH-receptor both directly by tyrosine kinase activation and indirectly by induction of insulin-like growth factor 1 (IGF-1) (7). Thus, the true effects of the multitudes of GH isoforms released in the different molecular weight fractions remain unclear or at least represent a composite effect based on fractional sizing analysis. IGF-I is a ubiquitous growth factor, also existing as several forms including free and binary/ternary complexes (8) and residing among several biocompartments (9), again with major roles in numerous metabolic and physiological functions (10). The systemic (e.g., hepatic) or local (e.g., skeletal muscle) release of IGF-I initiates its biological effects through several pathways, including PI3K-Akt-mTOR, and MAPK extracellular signal-regulated kinases (ERKs) (9, 11–13). Thus, the changes in these two superfamilies of hormones represent a powerful signaling network in human physiology.

A multitude of different studies (14–21) have provided insight into the influence of resistance- and endurance-based exercise on immunoreactive GH concentrations, yet far fewer studies have investigated the effects of exercise perturbations on GH molecular weight (MW) variants (19, 22). Regarding the latter, Wallace et al. observed that the increase, peak concentration, and disappearance differed among GH isoforms as a result of acute aerobic exercise in men (23). These included the 20- and 22-kDa isoforms, two of the earliest identified variants released from human pituitary cells (24). Hymer et al. were the first to describe acute increases in unfractionated GH and two MW fraction pools (<30, and 30–60 kDa) immediately following resistance exercise in untrained women (25), which was observed across several immunoreactive and immunofunctional assays. Contrasting these data, Kraemer et al. (26), examined the effects of acute resistance exercise intensity on GH isoforms stratified by MW in non-resistance exercise-trained women. Although no between-group differences in unfractionated GH were observed based on total work performed, differences in the smallest MW fraction (<30 kDa) were noted. Thus, distinct GH MW isoforms may respond differently than unfractionated GH, presenting a divergent response pattern by sex.

Previous studies have investigated the role of IGF-I concentrations in potentiating exercise-associated adaptations, as well as recovery from acute and chronic exercise (27–29). Similar to GH, acute exercise is an effective stimulus to alter and examine IGF-I profile responses. However, very little is known regarding the effects of acute exercise on fractionated IGF-I isoforms, as demonstrated with GH. For example, Durzynska et al. (30) characterized the different pre-pro forms of IGF-I peptide, which provided details within skeletal muscle-specific IGF-I MW isoforms. To our knowledge, this has yet to be examined in the systemic circulation.

Considering peptide heterogeneity and the dynamic interactions between GH and IGF-I, and the multiple biological adaptations attributed to these hormones, we proposed to examine their responses simultaneously in the blood to determine if diverse patterns exist reflective of the different temporal cybernetic release patterns in glands and tissues. By examining the blood biocompartment, this would enhance the understanding of the temporal time frames in men and women for the patterned responses of different GH and IGF-I MW isoforms, thus supporting further targeted investigations. Therefore, the purpose of this investigation was to determine if GH and IGF-I MW isoform responses to acute resistance exercise were due to specific MW isoforms in sex-independent or -dependent manners. We hypothesized that acute resistance exercise would drive sex-specific responses differently among GH and IGF-I family constituents (e.g., MW isoforms).

## Materials and Methods

### Subjects

Healthy, college-aged, men (*n* = 10; age: 28 ± 5 y, height: 171.9 ± 5.1 cm, weight: 86.2 ± 12.7 kg, BMI: 29.2 ± 4.5 kg·m^−2^), and women (*n* = 10; age: 21 ± 2 y, height: 165.4 ± 5.8 cm, weight: 67.1 ± 11.0 kg, BMI: 24.5 ± 3.3 kg·m^−2^) participated in this study. The volunteers had all experimental methods explained to them, and only participated after giving their free and voluntary written informed consent in accordance with the Declaration of Helsinki. All were screened *via* a health history examination, a physical examination by a physician, and were excluded if they had any conditions and/or taking any medications known to affect hormonal responses. All women were interviewed by the screening physician and deemed to have been eumenorrheic (having cycle lengths between 28 and 32 days for the previous several months leading up to the study) and not taking oral contraceptives. The women from this study were part of a broader longitudinal study (31), during which they were scheduled to complete multiple acute exercise trials during the same relative phase of their menstrual cycle. Attempts were made to have them complete their initial exercise trial as close to the early follicular phase as possible. We did not measure sex hormones to standardize timing, however, and only relied on verbal reports with regard to their menstrual cycle status/timing. Participants were untrained, and performed <3 exercise sessions per week for at least 6 months before the study, and no subjects were currently serving in the active duty military population. Sample sizes were determined using power calculations from previous studies that the authors have conducted in similar populations and protocols, which were primarily based on expected variability in IGF-I (typically less robust exercise-induced responses than GH), where 10 subjects per group was expected to yield 80% statistical power. All methods were reviewed and approved by the Human Use Review Committee of the U.S. Army Research Institute of Environmental Medicine. The investigators adhered to the policies for the protection of human subjects as prescribed in Army Regulation (AR) 70–25, and the research was conducted in adherence with the provisions of 32 CFR Part 219.

### Acute Resistance Exercise Test

Following a 10 h overnight fast, and refraining from strenuous exercise for 48 h, subjects were asked to report to the laboratory in the morning to perform an acute resistance exercise test (ARET). All ARET sessions took place between 600 and 1200 h in order to standardize the time of the perturbation. The ARET was chosen due to previous success in subject tolerance and the ability to perturb the hormonal milieu, as described elsewhere (25, 32). Briefly, the ARET was comprised of 6 sets of the individual's 10 repetition maximum (6 × 10-RM) squat, separated by 2-min inter-set recovery periods. The initial 10-RM weight was ~75% of the subject's 1-RM measured during pre-experimental testing sessions at least 48 h prior. The goal of each subsequent set was a 10-RM load, where the load was adjusted as needed to facilitate completion of 10 full range of motion repetitions with good form.

### Blood Sampling and Handling

Prior to the ARET, a venous catheter was inserted in a forearm vein with a saline lock to maintain catheter patency. Subjects had venous blood obtained before (Pre), after 3 sets (Mid), immediately post (Post), as well as 15-min (+15) and 30-min (+30) following the ARET. Blood samples (~7 mL) collected into SST vacutainers (BD, Franklin Lakes, NJ), clotted at room temperature for 30-min, and then centrifuged at 1,500 × *g* for 20-min. Serum aliquots were frozen and stored at −80°C until High Performance Liquid Chromatography (HPLC) processing and subsequent analyses were performed.

### Serum Fractionation

**Figure 1:**
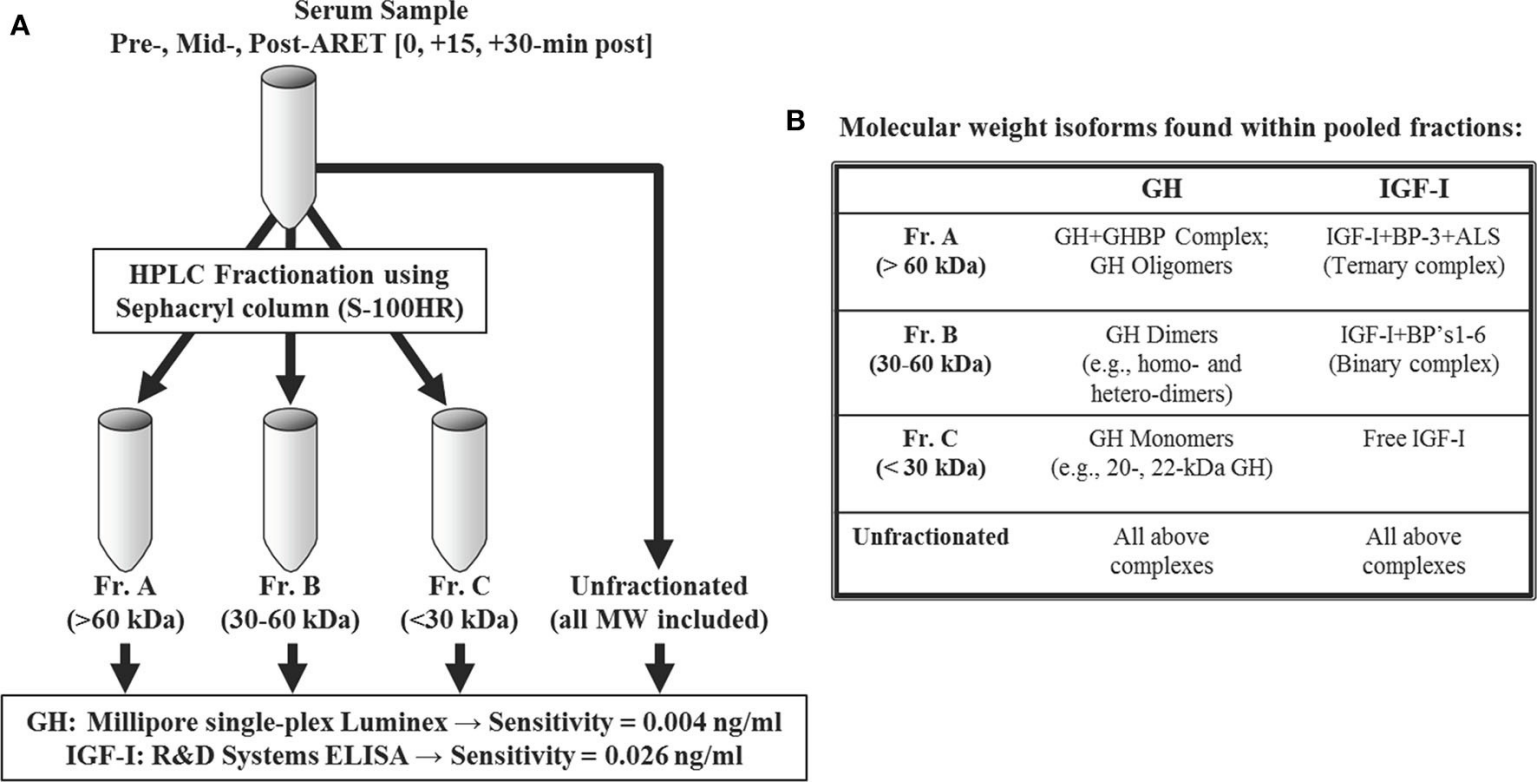
Size-excluded proteins via HPLC were pooled into three molecular weight (MW) ranges **(A)**. Details and examples of the corresponding MW isoforms expected to be found among fraction pools are provided in **(B)**. An additional aliquot was left unfractionated for recovery purposes. See Materials and Methods for immunoassay details.

At each time point noted above, serum was processed using HPLC and Sephacryl gel filtration columns [S-100HR sizing column (26 mm ID); GE Healthcare Bio-Sciences, Pittsburgh, PA] employing similar methods described previously (25). Briefly, the gel columns were calibrated with a MW standards kit (Pharmacia, Uppsala, Sweden), and proteins from each processed serum sample were eluted from the column based on size. Resultant collection tubes were subsequently pooled into 3 MW fraction ranges corresponding to >60 kDa (Fr.A), 30–60 kDa (Fr.B), and <30 kDa (Fr.C). Fractionated MW cut-off values were chosen to represent specific isoforms within the circulating GH-IGF-I axis, highlighting the heterogeneous nature of these related hormones (Figure 1).

### Immunoassays

All samples were run in duplicate; however, within-subject (fractionated and unfractionated) samples were run in the same plate to minimize inter-assay variability, with both intra- and inter-assay CV's <10 % for the GH and IGF-I assays. For GH analysis, unfractionated and fractionated serum samples from each time point were analyzed using a commercially-available bead-based human GH fluorescence assay (Millipore, Billerica, MA) with a reported sensitivity of 0.004 ng/mL. Concentrations were determined using a commercially available immunoassay platform (Luminex200; LuminexCorp, Austin, TX), and fluorescence values were quantified using Masterplex QT, v2.5 (Hitachi, San Bruno, CA). For IGF-I analysis, unfractionated and fractionated samples were analyzed using a commercially-available ELISA (DG-100; R&D Systems, Minneapolis, MN), with a reported sensitivity of 0.026 ng/mL. Absorbance values were quantified on a Dynex MRX Revelation absorbance reader (Dynex Technologies, Chantilly, VA). It should be noted that with the low abundance of circulating free IGF-I (e.g., IGF-I molecules measured in Fr.C) several samples failed to meet the assay sensitivity. In such cases, values were reported asthe lowest concentration detectable in the assay (e.g., assay sensitivity).

### Data Analysis

GH and IGF-I data were checked for normality with regard to fractions and time within sex using a Shapiro-Wilk test, and the majority were found to be normally distributed indicating that parametric statistics were appropriate. Subsequently, a 2-way (fraction × time) ANOVA with repeated measures (RM-ANOVA) with all subjects combined was used to determine whether GH or IGF-I responses to exercise were due to MW isoforms independent of sex. To answer the sex-dependent heterogeneity questions, a 3-way (fraction × time × sex) RM-ANOVA model for the interaction term was utilized. Subsequently, within-fraction 2-way (sex × time) RM-ANOVA models were run after identifying the significant 3-way interaction. When the RM-ANOVA detected a significant *F*-ratio, *post-hoc* analysis (least significant difference) was used to determine statistical differences for within- and between-subject factors. All values are expressed as mean ± SD, and α was ≤ 0.05, and statistical analyses were performed on SPSS version 21 (IBM, Armonk, NY).

## Results

In this study, we examined the total MW content of the fractionated serum samples via HPLC for both GH and IGF-I to determine hierarchical placements and relationships as they relate to sex. We also examined the unfractionated immunoreactivity in each of these samples to determine if the fractions were consistent with known antibody reactivity shown in prior work, and to determine if the patterns of responses to acute resistance exercise were similar between men and women with the desire to gain more information and understanding of this important metabolic and anabolic hormonal axis.

### GH

#### Fractionated GH via HPLC

We observed a main effect (*p* ≤ 0.01) on all GH fractions across time and sex, demonstrating differences among the abundance of all fractions in unexercised adults, and independent of sex where Fr.C (<30 kDa; 14.3 ± 11.5 ng) > Fr.A (>60 kDa; 9.6 ± 9.1 ng) > Fr.B (30–60 kDa; 5.2 ± 4.1 ng). There was a significant fraction × time interaction (*p* ≤ 0.01) effect observed on the exercise-induced GH responses, independent of sex. In response to exercise, all GH isoforms increased over baseline values (*p* ≤ 0.01), and significant differences were observed between all fractions (*p* ≤ 0.03) through +30-min of recovery.

**Figure 2:**
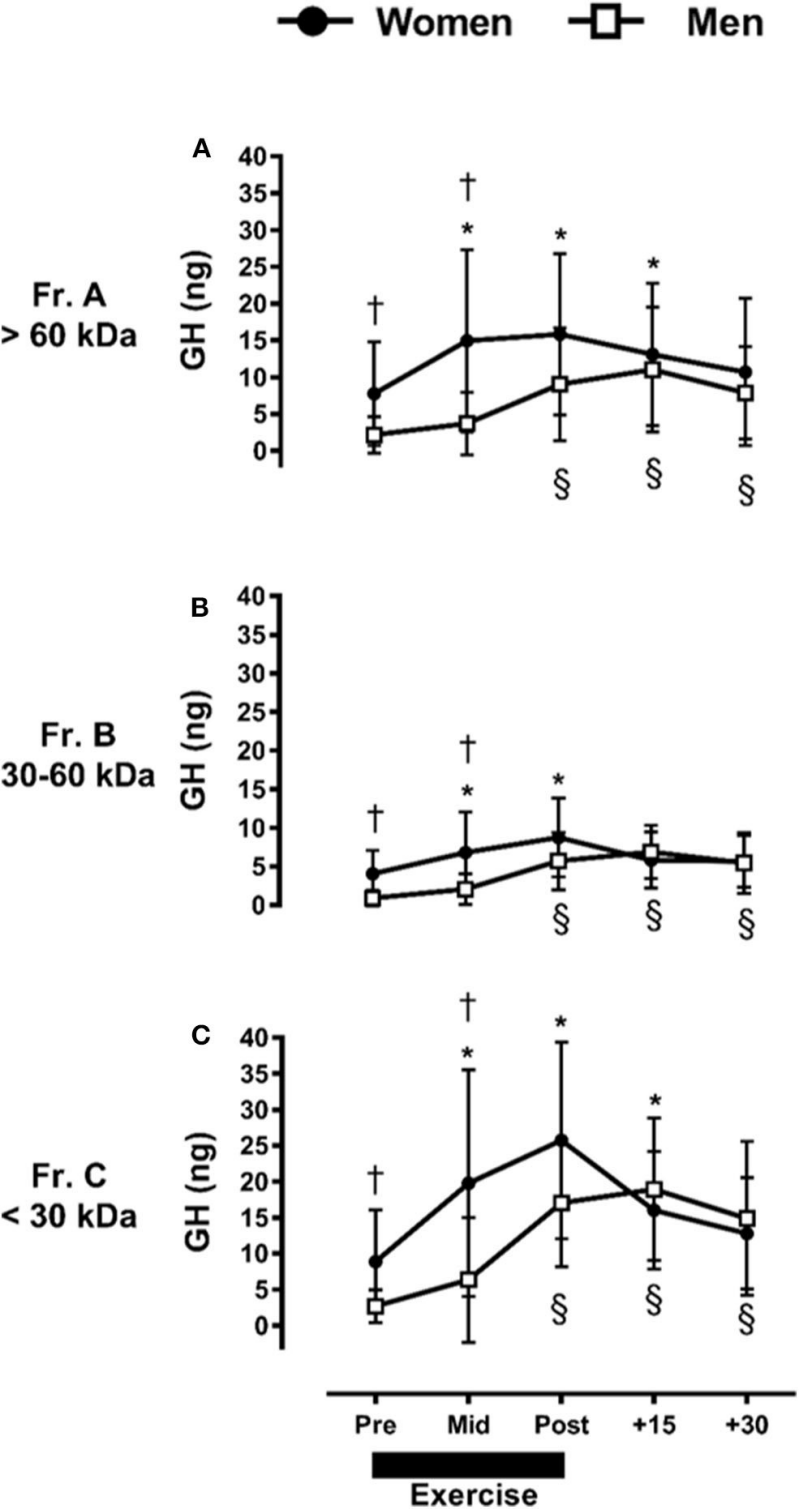
Sex-dependent exercise-induced responses in GH separated by MW size [Fractions (A–C) presented in **(A–C)**, respectively]. *P ≤ 0.01 vs. Pre-exercise within women; ^§^*P* ≤ 0.01 vs. Pre-exercise within men. ^†^ *P* ≤ 0.03 vs. men at time point within fraction.

We further identified a significant interaction between fraction, time, and sex. For instance, women had a greater abundance of Fr.A GH compared to men Pre- and Mid-exercise (*p* ≤ 0.03). Within women, resistance exercise-induced increases in Fr.A GH (*P* ≤ 0.03) represented by a baseline-to-peak 2.0 fold-change immediately post-exercise. Within men, Fr.A GH did not increase above baseline until post-exercise (*p* ≤ 0.01), with a baseline-to-peak 5.2 fold-change +15-min post-exercise, and remained elevated through +30-min of recovery (*p* ≤ 0.01) (Figure 2A). Fr.B isoforms were also higher in women than men (*p* ≤ 0.02) through mid-exercise. In women, Fr.B GH was elevated with exercise (*p* ≤ 0.01), with a baseline-to-peak 2.2 fold-change immediately post-exercise, and returned to baseline (*P* > 0.12) within +15-min of recovery. Men demonstrated a delayed increase (*P* ≤ 0.01) in Fr.B GH isoforms, where Fr.B GH was represented by a baseline-to-peak 7.7 fold-change +15-min post-exercise and remained elevated immediately post-exercise through +30-min of recovery (Figure 2B). Fr.C GH isoforms were also higher in women than men at Pre- and Mid-exercise (*P* ≤ 0.03). More specifically, Fr.C GH isoforms in women were elevated above baseline, represented by a baseline-to-peak 2.9 fold-change immediately post-exercise and remained elevated through +15-min following exercise. Men once again demonstrated a delayed and prolonged increase in Fr.C GH, represented by a baseline-to-peak 7.1 fold-change +15-min post-exercise and remained elevated through +30-min of recovery (*P* = 0.01) (Figure 2C).

When made relative to the total GH collected during fractionation, Fr.A GH ranged between 27 and 36%, Fr.B ranged between 15 and 20%, and Fr.C represented the highest abundance collected between 44 and 54%. Similar responses were observed for men and women (data not shown).

#### Unfractionated GH

Unlike MW-fractionated GH, unfractionated GH results did not present any main effects or interactions with sex as a factor. We only observed a significant main effect of time (data are not shown), which demonstrated that GH concentrations were increased Mid-exercise, and remained elevated through +30-min of recovery (*P* ≤ 0.03). Table 1 displays the relative recovery concentrations from fractionated compared to unfractionated GH.

**Table 1:** Recovery of GH and IGF-I molecular weight isoforms following HPLC fractionation compared to unfractionated.

	**Summed fractionated GH recovery**		**Summed fractionated IGF-I recovery**	
	**ng/mL**	**Fold difference**	**ng/mL**	**Fold difference**
**Pre**				
Women	41.28 ± 33.73	22.0	176.93 ± 67.66	1.05
Men	11.45 ± 11.23	49.8	171.06 ± 68.14	1.26
**Mid**				
Women	82.93 ± 64.84	13.3	190.12 ± 59.91	1.01
Men	24.23 ± 29.14	22.0	188.87 ± 69.9	1.31
**Post**				
Women	100.58 ± 57.10	11.4	190.50 ± 60.44	0.99
Men	63.63 ± 34.49	16.1	192.60 ± 74.49	1.39
**Post+15**				
Women	69.80 ± 40.57	12.8	171.30 ± 65.50	0.99
Men	73.69 ± 35.69	12.8	184.79 ± 60.90	1.36
**Post+30**				
Women	58.26 ± 40.18	16.3	176.66 ± 65.74	1.07
Men	56.35 ± 35.22	15.7	173.83 ± 51.79	1.36

*Summary of GH and IGF-I recovered following sample fractionation via HPLC Values are the mean ± SD (summed recovery) or mean only (fold difference) calculated from the individual recoveries. Recovery was determined as the sum of all collected protein fractions where concentration was corrected for HPLC dilution, and Fold difference was the sum of all fractions made relative to the unfractionated sample (ng/mL) at the same time point*.

### IGF-I

#### Fractionated IGF-I via HPLC

Similar to GH, we observed a main effect across all time points and sex, with differences among all IGF-I fractions collected (*P* ≤ 0.05), where Fr.A (>60 kDa; 76.6 ± 27.6 ng) > Fr.B (30–60 kDa; 11.6 ± 6.8 ng) > Fr.C (<30 kDa; 2.6 ± 3.6 ng) (*P* ≤ 0.01). Unlike GH, when IGF-I fractions are collapsed across sex, there were no significant exercise-induced changes over time within the three MW fraction pools (*P* > 0.05).

**Figure 3:**
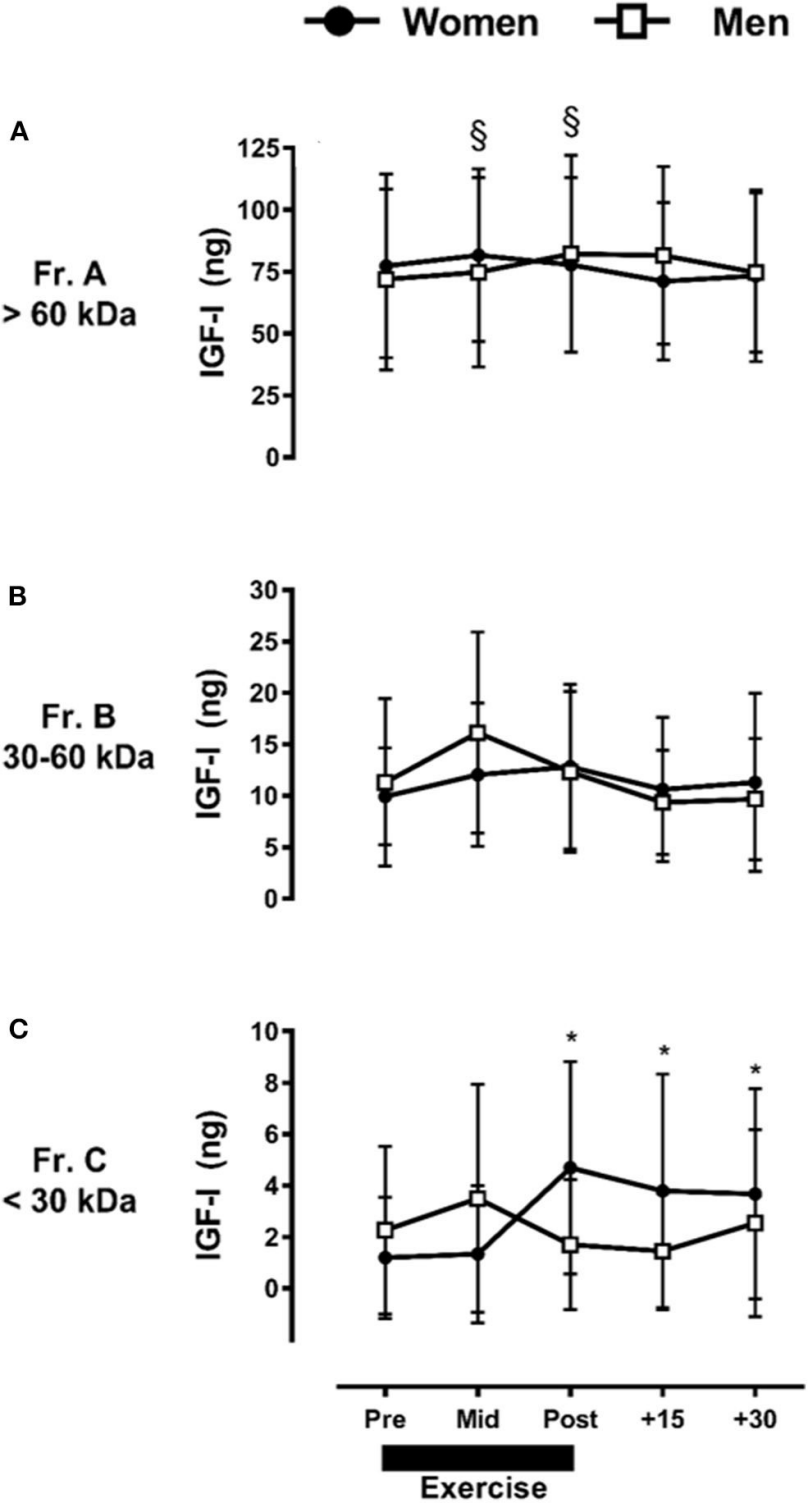
Sex-dependent exercise-induced responses in IGF-I separated by MW size [Fractions (A–C) presented in **(A–C)**, respectively]. Data are mean ± SD. *P ≤ 0.05 vs. Pre-exercise within women; ^§^*P* ≤ 0.05 vs. Pre-exercise within men.

Taking into account the observed interaction between fraction, time, and sex for IGF-I, the ARET induced a small but significant increase in Fr.A (*P* ≤ 0.05) only in men (Figure 3A), which represented a baseline-to-peak 1.1 fold-change immediately post-exercise. There were no exercise-induced changes at any time point in men or women for Fr.B IGF-I (*P* > 0.05) (Figure 3B). In contrast to Fr.A IGF-I, the ARET induced a significant increase in Fr.C IGF-I only in women, with a baseline-to-peak 3.9 fold change immediately post-exercise and remained elevated through +30-min of recovery (*P* ≤ 0.05) (Figure 3C).

When made relative to the total IGF-I collected with HPLC fractionation, Fr.A IGF-I ranged between 80 and 88%, Fr.B ranged between 10 and 17%, and Fr.C ranged between 1 and 5%. Similar responses were observed for men and women (data not shown).

#### Unfractionated IGF-I

We observed a main effect for sex between unfractionated IGF-I concentrations (women > men, *P* ≤ 0.05), although no significant differences between men and women were observed at any specific time point (data not shown). Table 1 displays recovery concentrations compared to unfractionated IGF-I.

## Discussion

Using HPLC fractionation of the serum along with immunoreactivity of the associated samples we observed sex-specific differences for different MW isoforms and an exercise-induced perturbation. The primary findings of the study were: (1) there were clear hierarchies in GH and IGF-I MW isoforms confirming the heterogeneous nature of this hormonal axis in men and women, and (2) fractionated GH and IGF-I responses to acute resistance exercise appear to be sex-dependent. These exercise-induced endocrine responses aid in the understanding of sex-specific hormonal control and underlying complex cascades partially mediating physiological adaptations from resistance exercise.

We observed a hierarchy among all GH MW fractions investigated via HPLC fractionation, which is in line with well-known GH heterogeneity, including nearly 100 different isoforms (5). From prior work, this hierarchy of difference is consistent and shows important internal validity of the HPLC analytical approach (33). In addition to 22-kDa GH, typically measured by most immunoassays, other GH MW isoforms include a 20-kDa mRNA splice variant, disulfide-linked homo- and hetero-dimers, glycosylated GH, high MW oligomers (up to pentamers), receptor-bound GH, and GH fragments (5, 32, 34–36). Distribution estimates of specific isoforms are known to vary extensively; however, the largest proportions are accounted for by monomeric 22- and 20-kDa GH (~70–85% of circulating GH), while heterodimeric and homodimeric GH account for ~10–20%, and oligomeric and GH bound to GHBP account for ~5–10% (5, 25, 36). Our results partially align with these pooled estimates, where the largest proportion of GH collected across all time points and sex in our study was Fr.C (<30 kDa), which includes the major circulating constituents: 20- and 22-kDa GH as well as segments and fragments of those monomers.

The second most abundant fraction collected in our study was Fr.A (>60 kDa), which would include high-molecular weight oligomers and GH-GHBP complexes. Although reports from both Baumann and Hymer suggest that Fr.A constituents are the least abundant (5, 25), it is possible that the subjects in our study had a higher abundance of oligomeric and/or GH-GHBP complexes in the circulation than previous estimates. In fact, higher GH concentrations in women might also be expected to lead to increased GHBP concentrations, given the dependency between GH and GHBP as demonstrated in female rats (37) and women (38). The investigation closest to ours in study design (25), which examined acute heavy resistance exercise in women, presented data within reasonable proximity to our Fr.C immunoreactive GH estimates (having accounted for ~50% of GH collected across all time points examined). This further suggests that the 191 amino acid, 22-kDa GH isoform detected by most GH immunoassays most likely accounted for the majority of our study's most abundant MW fraction, Fr. C (<30 kDa).

Men and women experienced exercise-induced increases among all GH MW isoform pools, but these responses were delayed and sustained longer in men. Our findings corroborate a recent study that demonstrated dimeric GH was higher in women, but sustained longer into recovery in men following resistance exercise (39). Additionally, Wallace et al. reported that a pituitary-derived GH assay (22-, 20-kDa, and other modified GH forms), as well as an exclusive 20-kDa assay, demonstrated an extended disappearance half-time following acute aerobic exercise in men (23). Those authors pointed out that their pituitary GH assay had a high affinity for dimeric GH, the presence of which could also help to explain prolonged elevations. In the context of our study, we observed sustained increases in all three GH fraction pools, which would cover monomeric 20-kDa GH, homo- and hetero-dimeric 20- and 22-kDa GH, GH-GHBP and oligomeric GH complexes, all of which possess increased half-lives when compared to monomeric 22-kDa GH (23, 40). We speculate that these sex-specific alterations in GH responses could relate to sex differences in GH's hypothalamic stimuli such as GHRH or somatostatin, leading to or inhibiting its pituitary release, respectively, and is likely under control of one or more sex steroid neuroendocrine loci including both estrogen and testosterone (41).

IGF-I MW fractions displayed clear differences across all time points and sex (main fraction effect) using HPLC fractionation, demonstrating a similar isoform hierarchy as observed with GH. Accordingly, we noted that the largest abundance of assayed IGF-I resided in Fr.A (>60 kDa), followed by Fr.B (30–60 kDa), and lastly by Fr.C (<30 kDa). These findings correspond with prior estimates of IGF-I existing primarily in ternary complexes (≥ 75%), followed by binary complexes (~20–25%) and finally in free form (<1%) (8). Similar to GH half-life extension through interactions with its binding protein, GHBP (40), the half-life of IGF-I complexes are altered depending on the isoform. Guler et al. (42) estimated that the half-life of the 150-kDa ternary IGF-complex (IGF-I+BP-3+ALS) is markedly increased (e.g., 12–15 h) over that of either binary-complexed (IGF-I + one of 6 BPs) (e.g., 20–30 min) or free-IGF-I (e.g., 10–12 min). Given our observation that the most abundant isoform pool was Fr.A (>60 kDa), it is reasonable to expect that IGF-I half-life would be extended considerably through the majority of IGF-I being sequestered in this MW fraction pool. The second largest abundance of IGF-I components was observed in Fr.B. Combined with Fr.A isoforms, these two MW pools (binary/ternary complexed IGF-I) accounted for ~97% of measured IGF-I.

We did not detect acute exercise-induced alterations in IGF-I isoforms over time when our data were collapsed by sex; however, a significant main effect of sex (women > men) was observed across all IGF-I MW fraction pools, and significant increases were apparent in two of three IGF-I MW fractions in a sex-dependent manner (Fractions A, C, Figures 3A,C, respectively). Thus, we only observed differences between the isoforms or over time within specific isoforms when sex was taken into account.

Regarding the sex-dependent IGF-I system exercise effects, we observed a significant increase in Fr.A (>60 kDa) with resistance exercise only in men, which indicates that only larger MW IGF-I isoforms (e.g., ternary complexes) were increased in men. Alternatively, we observed that Fr.C (<30 kDa) IGF-I molecules were increased following exercise and remained elevated throughout 30 min of recovery only in women. To our knowledge, we are the first to present this sexual dimorphism in the IGF-I system. Although we are unable to fully explain these observations other than lending further credence to the regulatory complexity provided through the family of 6 IGFBPs (8), it is possible that alterations in the IGFBP's affinity for IGF-I are also likely sexually-dimorphic, leading to our observed divergent responses in men and women.

There is a lack of congruence in the literature with the acute IGF-I response to exercise. This is in stark contrast to exercise-induced GH responses, and it is possible some of our limitations noted below may have led to some of these differences. With regard to GH, we recognize that using gel fractionation to create a <30 kDa pool limits our ability to distinguish 20- vs. 22-kDa GH; however, discerning between these two specific isoforms was not a main focus of the current investigation. With regard to IGF-I, we cannot differentiate between locally vs. systemically-derived IGF-I. For instance, local IGF has emerged as a contributor to the adaptations of exercise training; however, researchers have questioned the functional importance of IGF-I to post-natal skeletal muscle growth resulting from physical exercise (43–45). Further, our observation of heightened Fr.C IGF-I (<30 kDa) during and following exercise in women vs. men should be interpreted with caution, as there were several samples that did not meet the sensitivity of the assay employed. Of the total number of samples in Fr.C, only 43% of all samples met the sensitivity of the assay, and it should be noted that this phenomenon occurred more frequently in women (48% of samples) than in men (38% of samples). Also, more samples in post-exercise (0, +15, and +30 min post) met the assay sensitivity, and once again this was observed more frequently in women (63%) than in men (37%). Since free/unbound IGF-I demonstrates the lowest abundance among the IGF-I MW variants (accounting for <1% of all IGF-I) (8), and given sample dilution using HPLC fractionation, this was not a completely surprising observation. Finally, we did not specifically measure sex steroids and only relied on verbal reports of menstrual cycle timing when the participants performed the acute exercise test. Given that GH responses can vary directly (and vary indirectly via negative feedback from IGF-I) according to estrogen concentrations (41), it may be important to measure sex steroid concentrations concomitantly with systemic measures of the GH-IGF-I axis to more adequately characterize these responses. Future research should aim to confirm our findings given the above limitations.

Finally, while the sex-specific physiological significance of our findings are unclear, it is possible that the differential isoform responses observed currently may help to explain phenotype outcomes resulting from chronic exercise training. For instance, different exercise training regimens result in divergent tissue and organ adaptations (e.g., metabolic adaptations, cardiovascular improvements, bone and muscle tissue accumulation, etc.), which are clearly linked to the mode of training. Previous data suggested that chronic resistance training increased the GH bioactivity (as measured in the tibial line bioassay) among all three MW fractions in college-aged women, despite no consistent changes observed in immunoreactive GH (46). This has not been examined in long-term aerobic exercise training or in men to our knowledge, which could provide greater support to this speculation. Likewise, conducting a future similar study of IGF-I examined across MW isoforms and different exercise regimens coupled with a bioactive assay [e.g., kinase receptor activation (KIRA)] (47) could eluciate additional mechanisms by which this hormonal axis contrbutes to sex- and mode-specific chronic exercise-induced adaptations.

In conclusion, we observed sexually-dimorphic patterns in the GH and IGF-I hormonal axis. With regard to GH, women had higher abundances of GH in all three pooled fractions before and during exercise, but men experienced a delayed but sustained appearance of all three GH MW fractions following exercise and into recovery. Within the IGF-I family, men experienced acute increases in Fr.A (largest MW isoform pool mostly accounted for by ternary complexed IGF-I), while women experienced acute and sustained increases in Fr.C (smallest MW isoform pool mostly accounted for by free/unbound IGF-I). Once again, the changes in Fr.C IGF-I may have to be confirmed in future investigations using more sensitive techniques. We believe that the observed changes in GH and IGF-I MW isoform distributions are among factors explaining sex-specific phenotypic outcomes and metabolic alterations resulting from exercise.

## Data Availability Statement

Data are property of the U.S. Government and are only available through approved data sharing agreements between the government and individual institutions.

## Ethics Statement

The studies involving human participants were reviewed and approved by Human Use Review Committee U.S. Army Research Institute of Environmental Medicine. The patients/participants provided their written informed consent to participate in this study.

## Author Contributions

JP, JS, WK, WH, and BN contributed to the conception and design of the study. JA, JS, KR, WK, and BN contributed to the acute resistance exercise data and sample collection. JP, JA, JS, KR, and WH contributed to the HPLC fractionation methods and/or laboratory assays. JP and BM conducted the statistical analyses and wrote the initial draft of the manuscript. All authors contributed to the manuscript revision, read, and approved the submitted version.

## Conflict of Interest

The authors declare that the research was conducted in the absence of any commercial or financial relationships that could be construed as a potential conflict of interest.
